# Rethinking criminal profiling through cognitive artificial intelligence

**DOI:** 10.3389/frai.2026.1824060

**Published:** 2026-05-08

**Authors:** Jorge Buele, Davis Miranda-Toapanta, David Rojas-Cañizares

**Affiliations:** Centro de Investigación en Mecatrónica y Sistemas Interactivos (MIST), Facultad de Ingenierías, Universidad Tecnológica Indoamérica, Ambato, Ecuador

**Keywords:** artificial intelligence, cognitive computing, explainable AI, forensic AI, pattern detection

## Abstract

**Introduction:**

Traditional criminal investigation often struggles to integrate dispersed and heterogeneous information, delaying the identification of serial or escalating patterns. Advances in artificial intelligence (AI) and cognitive computing offer data-driven approaches for cross-source correlation and temporal anomaly detection.

**Methods:**

A focused narrative review of peer-reviewed literature on AI applications in forensic analysis, pattern detection, and investigative support was conducted using major multidisciplinary databases. Selected studies were synthesized into two analytical dimensions: evidence correlation and anomaly detection, and further examined through a retrospective case-based illustration.

**Results:**

AI-based approaches support the linkage of low-level traces with higher-level events, enabling structured reconstruction and large-scale pattern identification. Machine learning models integrate heterogeneous data into operational representations, achieving high predictive performance under controlled conditions, often reported above 90% accuracy. Statistical and learning-based methods also detect temporal compression, behavioral drift, and cross-source anomalies, revealing patterns that may remain fragmented under manual analysis. Retrospective examination of historically fragmented cases highlights longitudinal regularities, including shrinking inter-event intervals, increasing severity, and the accumulation of weak signals that become meaningful when analyzed jointly.

**Discussion:**

AI contributes primarily at a methodological level by enabling continuous integration and re-evaluation of investigative signals, supporting more data-informed and longitudinally grounded profiling practices. However, a gap persists between high performance in controlled settings and limited validation in real-world contexts. Future research should prioritize empirical benchmarking using operational datasets, the development of explainable and auditable systems, and governance frameworks ensuring transparent, accountable, and human-supervised deployment.

## 1 Introduction

Extreme violent crime has often been framed in moral and philosophical terms that seek to define “evil” in human behavior (Ellens, 2011). In investigative practice, however, effective action depends less on such abstractions than on recognizing concrete patterns within dispersed and imperfect information. Traditional criminal profiling relies on expert synthesis of crime scene and behavioral cues, and substantial research has identified neurodevelopmental and psychosocial risk factors in severe violent offenders (Allely et al., 2014), often through detailed psychobiographical reconstructions of individual cases (Fouché et al., 2015). Yet transforming this individual-level understanding into timely recognition of emerging criminal patterns remains primarily a human-driven task, exposed to bias, fatigue, and difficulty handling large, heterogeneous datasets.

Recent literature has formalized recurrent cognitive pitfalls in investigative work. Hidden decision biases, over commitment to early hypotheses, and paralysis in the face of unstructured information can lead investigators to systematically underuse available signals (Martens, 2011; Maarek and Darwish, 2023). In practice, these effects manifest as fragmented case handling, delayed detection of seriality, and inconsistent valuation of community warnings that appear minor when considered in isolation (Al Matrooshi, 2024).

Advances in cognitive artificial intelligence (AI) introduce an alternative paradigm centered on continuous correlation rather than episodic interpretation. In this study, cognitive AI is defined as the use of AI systems to support continuous, context-aware integration and interpretation of heterogeneous investigative data. Instead of constructing profiles primarily from expert intuition, data-driven systems can persistently aggregate events across time, locations, and sources, surfacing statistically significant regularities that may escape human sequential review patterns (Vimala et al., 2024). Importantly, this shift is methodological rather than sensational: the goal is not to “predict monsters,” but to detect abnormal trajectories, accelerations, and cross-case links within complex datasets.

Historical cases are often revisited in the literature to demonstrate how meaningful warning signals can exist for years without being analytically integrated. The Dahmer case (1978–1991) exemplifies this situation, not for the figure of the offender but for the accumulation of minor complaints, police contacts, and local disturbances that were handled as isolated incidents rather than as parts of an evolving pattern Retrospective analyses show a clear long-term escalation in frequency and severity of offences, illustrating how temporal acceleration, behavioral drift, and geographically concentrated anomalies can remain undetected when information is fragmented and assessed moment by moment (Aswathy and Indu, 2025).

Recent adjacent literature has demonstrated substantial progress in the use of AI for crime prediction, spatio-temporal forecasting, and digital forensic automation, but these lines of research have developed around analytical priorities different from those addressed here. Existing reviews highlight methodological diversity alongside persistent limitations in interpretability, evaluation consistency, and real-world applicability (Dakalbab et al., 2022; Du and Ding, 2023). In parallel, digital forensics research has emphasized the evaluation and admissibility of AI-assisted evidence mining, while broader syntheses confirm ongoing advances in the field (El-Latif et al., 2025). However, these contributions leave underexplored the intersection between cognitive AI as defined in this study, fragmented investigative records, longitudinal escalation signals, and explainable support for criminal pattern recognition.

Against this background, contemporary forensic AI approaches increasingly address fragmentation through evidence correlation, anomaly detection, and multi-source signal aggregation. This mini review examines how such methods contribute to the integration and interpretation of dispersed investigative data, and discusses their implications for explainability, human oversight, and responsible use in practice.

## 2 Materials and methods

### 2.1 Literature search strategy

An exploratory search was performed in multidisciplinary and technical databases, including Scopus, Web of Science, IEEE Xplore, and PubMed. The search combined terms related to artificial intelligence and cognitive computing with concepts from digital forensics and criminal investigation. Typical query strings included combinations of “artificial intelligence,” “machine learning,” and “cognitive computing” with terms such as “forensic analysis,” “crime pattern detection”, “criminal investigation”, “automated forensic examiner”, and “explainable AI.” Emphasis was placed on peer-reviewed journal articles and conference proceedings in computer science, digital forensics, and applied AI. Highly cited or foundational papers were incorporated where necessary to clarify key methodological ideas or widely used frameworks.

### 2.2 Selection criteria

Publications were selected when they provided explicit methodological or technical descriptions of how AI systems perform evidence correlation, anomaly detection, behavioral pattern modelling, or investigative decision support. Additional consideration was given to works discussing transparency, bias, and accountability in AI-assisted decision processes. Sources were excluded when they consisted solely of narrative or journalistic accounts of crimes without analytical or technical contribution, when they addressed psychological profiling without computational components, or when they treated AI applications in domains unrelated to transferable investigative or forensic methods. Historical criminological analyses were used only to document timelines, escalation dynamics, and instances of investigative fragmentation in a representative serial crime case, serving as contextual reference points rather than primary technical evidence.

### 2.3 Analytical approach

The selected literature was analyzed through thematic synthesis. Reported methods and findings were organized into two functional dimensions: automated forensic correlation and reconstruction of evidence across heterogeneous data sources, and cognitive pattern modelling for anomaly detection and decision support. A single well-documented serial crime case was revisited retrospectively to illustrate how dispersed signals and delayed pattern recognition can occur when information remains fragmented. This comparison related the documented computational capabilities to a real investigative context in which relevant signals existed but were not analytically integrated at the time. Ethical, legal, and explainability considerations were not treated as empirical results but were derived in the discussion as implications of deploying such AI-based cognitive systems in practice.

## 3 Results

### 3.1 Artificial intelligence for evidence correlation and reconstruction

The reviewed literature describes artificial intelligence as a mechanism for large-scale correlation of dispersed forensic artifacts and the reconstruction of event sequences. Rather than analyzing traces in isolation, AI-based frameworks integrate high-level criminal events with low-level digital and contextual data across multiple sources, enabling more comprehensive analytical processes.

The concept of advanced automated forensic systems has been discussed as an approach to support iterative evidence processing instead of static, isolated findings (Michelet et al., 2023). Once relevant artifacts are identified, subsequent tasks such as extraction, correlation, and timeline reconstruction can be performed, generating structured representations that improve analytical consistency and reduce manual workload. This reflects broader developments in digital forensics driven by increasing data volumes and investigative backlogs.

Machine learning and deep learning models further enable the integration of heterogeneous crime-related data into structured representations for classification and prediction. Under controlled conditions, these models achieve high predictive performance, with accuracy levels reaching up to 97% depending on the dataset, features, and model architecture (Ngoge et al., 2024; Lu et al., 2025). Recent advances incorporate hybrid architectures combining Transformer-based global dependency modeling with convolutional neural networks for local feature extraction, facilitating the identification of spatial, temporal, and contextual patterns that are difficult to detect through manual analysis.

Additionally, AI-based approaches support the identification of behavioral patterns and indicators of technical sophistication through the analysis of communication patterns and interaction dynamics. By integrating large-scale digital data, including social media and online communications, these systems expand the analytical scope of investigations and contribute to more structured forensic processes (Mateo-Fernandez and Osa-Subtil, 2024). Detailed algorithmic configurations, application domains, and quantitative performance metrics of the reviewed studies are summarized in Table 1.

**Table 1:** Summary of AI methods, algorithms, and quantitative performance in reviewed studies.

Study	AI/analytical approach	Model/method	Main task	Evidence base	Evaluation/evidence	Main contribution
Michelet et al. (2023)	Digital forensic automation	Conceptual and interview-based framework	Define and classify automation in digital forensics	Literature review + 3 practitioner interviews	Conceptual and practitioner-informed analysis; no benchmark metrics reported	Proposes a definition of automation and distinguishes basic automation, automated tasks, and autonomous investigation
Ngoge et al. (2024)	Machine learning for crime prediction	Random Forest, Naive Bayes, SVM, Decision Tree, KNN	Crime classification and spatial–temporal mapping	Real-world Nairobi crime dataset (2059 instances)	Comparative model evaluation; Random Forest reached 97% accuracy	Shows that ML can support crime category prediction and location-based mapping, with RF as best-performing model
Lu et al. (2025)	Lightweight deep learning for crime pattern recognition	Transformer + shallow CNN with simulated annealing sparsity (LCRNet)	Crime pattern recognition / classification	Real-world LAPD dataset + cross-dataset testing	Accuracy, precision, recall, F1, FLOPs, ablation, and generalizability tests; 97.76% accuracy, 97.75% F1	Demonstrates a lightweight yet accurate architecture with reduced computational cost and cross-dataset generalization
Mateo-Fernandez and Osa-Subtil (2024)	AI-supported digital forensic psychology	Narrative review of AI, social media analysis, and interdisciplinary methods	Criminal profiling in digital environments	Review article drawing on prior studies	Narrative/theoretical synthesis; no direct system validation reported	Argues that AI and social media analysis can improve profiling, early threat detection, and interdisciplinary forensic assessment
Kuzmanov (2025)	AI-augmented profiling	Conceptual and critical analysis	Profiling, cognitive limitations, and human-AI collaboration	Theoretical/conceptual paper	Theoretical argument; no empirical benchmark metrics reported	Identifies cognitive biases in traditional profiling and advocates a human-in-the-loop “centaur” model with explainability
Lankford and Hayes (2022)	Longitudinal behavioral analysis	Retrospective case-based temporal interpretation	Escalation/temporal progression in serial offending	Case-based/retrospective analysis	Qualitative-temporal evidence rather than benchmark metrics	Supports the interpretation of non-linear escalation and interval compression in serial offending

CNN, convolutional neural network; LCRNet, lightweight crime recognition network; ML, machine learning; SVM, support vector machine; KNN, k-nearest neighbors; RF, Random Forest; FLOPs, floating point operations (computational cost metric).

### 3.2 Cognitive pattern modelling and investigative prioritization

Beyond artifact correlation, AI-augmented approaches enable the identification of behavioral patterns and deviations across large datasets, supporting investigative prioritization through the detection of anomalies and emerging risk signals (Kuzmanov, 2025). These systems can reveal patterns such as escalation, recurrence, and convergence that may remain fragmented under manual analysis, allowing the recognition of shifts in activity and evolving modus operandi.

From a technical perspective, these processes rely on classification, clustering, and predictive modeling techniques, evaluated through metrics such as accuracy, precision, recall, and F1-score. By applying consistent statistical criteria, AI reduces dependence on subjective interpretation and functions as a cognitive support layer that surfaces reproducible, data-driven associations for human investigators to interpret rather than replace their judgment (AlFahdi et al., 2014).

### 3.3 Case-based illustration of detectable longitudinal patterns

Viewed retrospectively through a data-centric lens, the Dahmer case exhibits measurable longitudinal patterns that were not operationally integrated during the investigation. Crime timelines reveal an initial dormancy followed by progressive compression of inter-event intervals, culminating in multiple homicides within a short period in 1991. Analytically, this reflects a non-linear acceleration pattern rather than isolated incidents (Lankford and Hayes, 2022).

In parallel, documented behavior shows increasing severity over time, evolving toward more invasive and controlling practices. From a computational perspective, this progression can be interpreted as a directional drift in behavioral features rather than random fluctuation. Such escalation aligns with longitudinal trajectory models of serial offenders, where patterns emerge through cumulative changes rather than discrete events (Reid et al., 2019).

Multiple weak contextual signals were also present, including repeated neighbor complaints, minor police contacts, and geographically concentrated disappearances. While individually ambiguous, their aggregation forms a coherent cross-source anomaly. Their historical treatment as unrelated incidents illustrates how institutional and social factors contributed to signal fragmentation and risk underestimation (Mariya and Ramdane, 2024), a limitation that automated correlation frameworks aim to address.

While these patterns are identifiable in retrospective analysis, translating them into reliable real-time detection remains challenging. Models achieving high performance in controlled datasets, often exceeding 90% accuracy, do not necessarily generalize to complex investigative environments (Ngoge et al., 2024; Lu et al., 2025). Variability in data quality, feature distributions, and incomplete records can affect model stability and applicability, and despite advances in computational efficiency, real-world deployment remains insufficiently validated.

Episodes such as the 1991 street encounter, where an initial benign interpretation prevailed despite contradictory cues, illustrate the limits of isolated human judgment. In contrast, continuous data integration would allow iterative re-evaluation of such events, enabling emerging patterns to become progressively detectable and operationally relevant.

## 4 Discussion

The results indicate that relevant patterns were present but not analytically integrated: shrinking intervals between events, progressive behavioral intensification, and multiple weak signals dispersed across independent reports. What historically failed was less the absence of clues than the absence of mechanisms to continuously aggregate and re-evaluate them. In this light, the contribution of AI is primarily methodological rather than interpretative: it enables persistent longitudinal detection and cross-source correlation that human, case-by-case investigation may struggle to sustain over extended time spans and large datasets (Goh et al., 2025).

A key implication is not simply the detection of patterns, but their appropriate contextualization. Contemporary approaches in digital forensic psychology emphasize the identification of subtle behavioral changes, particularly in communication and interaction dynamics, as potential indicators of escalation or emerging risk (Mateo-Fernandez and Osa-Subtil, 2024). However, the analytical value of these signals depends on how they are interpreted within broader ethical, psychological, and epistemic frameworks.

Evaluation practices further complicate this landscape. While AI-based systems are commonly assessed using metrics such as precision, recall, and false positive rates, these criteria vary across domains. Crime-related applications often prioritize predictive performance, whereas digital forensics emphasizes evidentiary reliability, transparency, and admissibility (Solanke and Biasiotti, 2022). In addition, the notion of automation in forensic contexts remains inconsistently defined, reflecting fragmentation in terminology and evaluation approaches (Michelet et al., 2023). This suggests that benchmarking AI for investigative support should move beyond single-metric performance and incorporate detection capability, interpretability, and operational relevance.

### 4.1 Escalation as a computational signal

Describing the progression of crimes as “addiction-like” may be clinically suggestive (Lankford and Hayes, 2022), but for AI-supported analysis its relevance lies in measurable temporal dynamics rather than diagnostic interpretation. Cognitive systems can detect the contraction of inter-event intervals and the systematic increase of severity-related features over time.

These longitudinal structures can be formalized as statistical change signals relative to background crime patterns. Correlation and prediction models are capable of identifying such non-linear acceleration without requiring prior knowledge of the offender or motive, instead relying on deviations from learned baselines (Allam et al., 2021). This distinction is critical: machine outputs indicate atypical temporal structures, while their criminological or clinical interpretation remains a human responsibility.

An important research direction is the systematic benchmarking of temporal anomaly detection methods on historical crime series to evaluate how early statistically reliable signals could be identified under realistic conditions (Butt et al., 2021).

### 4.2 From isolated judgments to continuous integration

The case illustrates how decisive moments were historically evaluated in isolation and shaped by initial interpretations, without cumulative integration of signals across time and sources. AI-based systems address this limitation by enabling continuous updating of assessments as new data become available, supporting iterative linkage of artifacts within broader correlation structures (Cekic, 2024; AlFahdi et al., 2014).

This approach does not eliminate human bias, but may reduce the effects of fatigue and information fragmentation by maintaining previously identified anomalies within an active analytical context. While multimodal and behavioral signals can further enrich this process, their contribution should remain supportive and subject to validation. Robust field evaluation is required to assess their reliability and to prevent disproportionate influence of context-sensitive or culturally dependent cues (Durán et al., 2024).

### 4.3 Human–AI collaboration and explainability

The findings support a collaborative model in which AI systems contribute large-scale pattern detection while human investigators provide contextual interpretation, ethical judgment, and domain expertise (Kuzmanov, 2025). In this framework, the value of AI lies in surfacing statistically grounded signals, whereas meaning and action remain contingent on human evaluation.

This distinction is particularly relevant in contexts where certain signals have been historically under-recognized. Data-driven anomaly detection, including spatial–temporal clustering, may help reveal patterns that were previously overlooked; however, their usefulness depends on transparent and interpretable outputs (Mateo-Fernandez and Osa-Subtil, 2024). Without this, high predictive performance does not necessarily translate into reliable decision support.

Explainability therefore plays a central role in enabling critical inspection of AI-generated outputs. Investigators must be able to examine the variables and relationships underlying alerts in order to validate, contextualize, and, when necessary, challenge them (Singh et al., 2024). Beyond technical transparency, this supports procedural accountability and reduces the risk of reproducing historical biases. Future research should jointly evaluate detection performance and explanation quality, including how different forms of explanation influence investigative reasoning (Faqir, 2023).

### 4.4 Governance, error, and proportionality

While aggregating dispersed signals offers clear analytical advantages, its large-scale implementation introduces legal and ethical tensions. Continuous cross-source correlation entails extensive data collection and retention, which may conflict with privacy and data protection principles. When AI-supported outputs inform prioritization or intervention, errors carry asymmetric consequences: missed patterns may delay protective action, whereas false positives may unjustifiably direct investigative attention. Current legal frameworks provide limited guidance on how responsibility should be allocated among developers and operators in such contexts (Singh et al., 2024).

Accordingly, analytical capability should be matched by appropriate governance mechanisms. Practical deployment requires clearly defined thresholds for action, mandatory human oversight in consequential decisions, and traceable reasoning processes supported by explainable models. In addition, independent auditing and periodic impact assessments are necessary to monitor system behavior, assess unintended effects, and ensure alignment with legal and ethical standards throughout the system lifecycle (Laine et al., 2024).

### 4.5 Limitations

This mini review is subject to several limitations that should be considered when interpreting its conclusions. The literature search was restricted to academic publications in English, which facilitates comparability but may exclude relevant contributions from non-Anglophone contexts, particularly in fields such as criminology, digital forensics, and legal theory. As a result, the synthesis may reflect a predominantly Western perspective, potentially limiting the consideration of alternative conceptual or normative frameworks.

In addition, the case-based illustration is derived from secondary and retrospective analyses rather than primary investigative data. Consequently, interpretations of escalation patterns and systemic limitations depend on prior reconstructions, positioning this contribution as a conceptual reinterpretation rather than an empirical re-analysis. More broadly, as a focused mini review, this work prioritizes theoretical and methodological integration over exhaustive empirical validation. Although quantitative performance indicators reported in the literature are considered, the study does not include standardized benchmarking of algorithms nor validation using real-world investigative datasets. Therefore, the discussion should be understood as a prospective analytical framework that outlines potential capabilities and governance considerations, rather than as evidence of operational effectiveness in applied investigative settings.

## 5 Conclusion

This mini review indicates that artificial intelligence can operationalize the continuous integration and analysis of dispersed forensic information, enabling the detection of longitudinal and cross-source patterns that are difficult to identify through case-by-case investigation. Rather than introducing new forms of interpretative judgment, these systems extend analytical capacity by systematically linking heterogeneous data, reconstructing event sequences, and highlighting statistically relevant deviations across time and context. In doing so, they make visible structured patterns, such as escalation, recurrence, and convergence that may remain undetected when signals are processed in isolation, reframing criminal profiling as a process grounded in continuous data integration rather than episodic interpretation. At the same time, the findings suggest that these capabilities remain contingent on controlled conditions and are not yet consistently validated in real-world investigative environments. This limits their current role to analytical support rather than autonomous decision-making. Their effective use therefore depends on the ability to contextualize machine-generated outputs within human judgment, particularly when signals are ambiguous, incomplete, or socially sensitive, reinforcing a hybrid approach in which cognitive AI augments, rather than replaces, investigative reasoning.

Future work should focus on empirically evaluating temporal and cross-source detection methods using operational datasets, as well as validating behavioral and multimodal indicators under field conditions. In parallel, the development of explainable AI frameworks is essential to ensure that outputs are transparent, auditable, and suitable for decision-support contexts. Advancing this integration will also require governance approaches that combine human oversight, proportional data use, and independent auditing, ensuring that enhanced pattern detection is aligned with legal, ethical, and epistemic standards.

## Data Availability

No new data were created or analyzed in this study. Data supporting the findings of this review consist exclusively of previously published articles and publicly available institutional reports cited within the manuscript.
